# Telephone-Administered Cognitive Behavioral Therapy for Body Dysmorphic Disorder: Case Series

**DOI:** 10.3390/ijerph19127373

**Published:** 2022-06-16

**Authors:** Marie Drüge, Tanja Roth, Birgit Watzke

**Affiliations:** Department of Psychology, Clinical Psychology with Focus on Psychotherapy Research, University of Zurich, Binzmühlestrasse 14, 8050 Zurich, Switzerland; tanja.roth@uzh.ch (T.R.); b.watzke@psychologie.uzh.ch (B.W.)

**Keywords:** body dysmorphic disorder, case series, cognitive behavioral therapy, e-mental health, obsessive-compulsive spectrum disorders, telemedicine, telephone-administered therapy

## Abstract

Cognitive behavioral therapy is an effective treatment for body dysmorphic disorder (BDD), but many patients do not receive appropriate treatment due to several treatment barriers and psychosocial care structures. Low-threshold interventions, including those from the field of e-mental health, could improve access to psychotherapy. In addition to internet-administered therapy, telephone-administered therapy may reduce treatment barriers, especially during the COVID-19 pandemic. This article presents four case reports of the same treatment (12 weeks of telephone-administered cognitive behavioral therapy accompanied by a workbook) applied to patients with body dysmorphic disorder during the summer of 2020. Three patients who completed the treatment had clinically relevant reductions in body dysmorphic and depressive symptoms and improved insight. One patient did not complete the telephone-administered therapy because her symptoms worsened, and she needed a more intensive form of treatment. These findings encourage future studies on the efficacy and effectiveness of telephone-administered treatment for BDD and its role in stepped-care models.

## 1. Introduction

### 1.1. Body Dysmorphic Disorder

In recent decades, body dysmorphic disorder (BDD), defined as a distressing preoccupation with a perceived defect in one’s appearance that is not apparent to others and leads to time-consuming behaviors (such as applying/checking on make-up) and thought patterns [1], has attracted increasing attention. BDD is associated with a high burden of disease and may therefore cause psychosocial impairments (e.g., social isolation) and suicidal thoughts/behaviors [2]. The point prevalence of this disorder is 2.9% [3]. Comorbidities such as mood or anxiety disorders, as well as social phobia and substance-use disorders, are common and often occur as secondary problems [4]. In two-thirds of cases, the age at onset is during adolescence [5].

### 1.2. Treatment Options for BDD

If left untreated, BDD can be a severe and chronic disorder, which is unlikely to remit. Two forms of treatment have been found to be efficacious in initial meta-analytic review papers [6,7], namely cognitive behavioral therapy (CBT) and serotonin reuptake inhibitors (SSRIs). The National Institute for Health and Care Excellence (NICE) has developed the only clinical guidelines for BDD treatment [8] and recommends a combination treatment if necessary. CBT for BDD applies some of the core principles of CBT, such as psychoeducation, cognitive restructuring behavioral experiments targeting unrealistic negative thoughts, in vivo exposures with response prevention targeting perpetuating factors (e.g., safety and avoidance behavior) and relapse prevention [9,10]. Unfortunately, as little as 8% of individuals with BDD state receive CBT [11]. To improve the low treatment utilization patterns among individuals with BDD and to broaden the scope of low-threshold options, one research group has developed and tested (pilot study, randomized controlled trial, follow up) an internet-based CBT course for BDD (BDD-NET; [12,13,14,15]), and two other e-mental health approaches for BDD have also been developed recently [16,17]. Telemedicine has become more common in the treatment of obsessive-compulsive disorders (OCD) over the past three decades [18] and has been found to be effective in a recent metanalytic approach [19]. Interestingly, in addition to internet-based therapies, telephone-based therapies have also been shown to be effective [20], even if research has focused more on digital mental health approaches.

### 1.3. COVID-19 and BDD

The outbreak of COVID-19 had a large impact on everyday life, as well as on the delivery of psychotherapy; several studies have reported interrupted treatments or a change in modality toward tele-psychotherapy (e.g., [21]). Furthermore, studies have found increased severity of symptoms of anxiety or depression and body dysmorphic symptoms [22]. Drüge et al. [23] provided an overview of difficulties encountered during the COVID-19 pandemic and its containment measures in therapy and in the everyday lives of BDD patients. During the COVID-19 pandemic, standard face-to-face therapy was not possible due to social distancing measures. Moreover, some patients indicated that they would like to have remote treatment but did not want to receive remote video treatment due to video mirroring and screen reflections [23]. Thus, a telephone therapy course was developed in which the patients were therapeutically supported by workbooks and weekly telephone calls.

Since these are case reports, no formal research questions were established. Nevertheless, this work aims to explore the extent to which the presented patients benefitted from BDD-TEL: how does BDD symptomatology change pre- to post-treatment? How does the depressive symptomatology change pre- to post-treatment? How does insight change pre- to post-treatment? Are there are any particular difficulties (e.g., during implementation) or obstacles?

## 2. Materials and Methods

### 2.1. Measures

Diagnoses were established by the Structured Clinical Interview for DSM-V (SCID-V, [24]). Further assessments involved measures such as the Patient Health Questionnaire-9 (PHQ-9, [25]) and the General Anxiety Symptoms-7 (GAD-7, [26]). The diagnostic process involved specific measures for BDD (“Fragebogen Körperdysmorpher Symptome”, questionnaire for BDD symptoms, FKS, [27]) and the Brown Assessment of Beliefs Scale (BABS; [28]) was used to assess insight.

### 2.2. Treatment: Telephone-Administered Cognitive Behavioral Therapy for BDD (BDD-TEL)

BDD-TEL consisted of a workbook (“Being able to live with the mirror”) in German and weekly telephone-administered sessions.

The workbook (“Being able to live with the mirror”) was based on BDD-NET [12,13,14,15], adapted to suit the format and procedures of a telephone-based intervention and was developed for treating depression in prior research projects [29,30]. Similar to BDD-NET, it contained eight modules consisting of readings, worksheet exercises, and homework questions (see Table 1). Modules 2–8 started with a page that listed things to do and to prepare for the next telephone session, as in the telephone-administered cognitive-behavioral program for depression [29,30].

There were 8 modules, delivered over 12 sessions, spanning 3 to 5 months. As soon as the patient and therapist agreed that they had completed the content of one module, they moved to the next. The first two modules focused on psychoeducation and generated an individualized model of BDD; the third module focused on unhelpful thoughts and cognitive reconstruction. The fourth and fifth modules consisted of exposure therapy and response prevention, and the sixth module focused on life values. The last two modules provided help for difficulties and setbacks and developed personalized strategies for prevention. In total, the program spanned 3 to 5 months and included a preliminary face-to-face session, as well as 360 min of telephone-administered psychotherapy delivered over 12 sessions by an experienced and licensed psychotherapist who was involved in the development of the workbook. The structure of the clinic included monthly supervision by experienced psychiatrists and psychotherapists.

### 2.3. Procedure

For two patients, the first contact to the outpatient clinic was via telephone concerning a need for BDD treatment during the first wave of COVID-19; the two other patients had received outpatient treatment before, focusing on comorbid conditions. The two returning patients sought BDD-specific treatment, as their prior treatment focused on comorbid conditions, but their BDD symptoms increased during the lockdown. In 2020, little was known about COVID-19; safety measures were taken seriously, and many treatments were interrupted or their modalities changed to tele-psychotherapy. Therefore, most of the procedures were assessed without face-to-face contact. All patients were provided with informative materials about BDD-TEL, and a telephone call was used to address further questions. Written informed consent was obtained before the initial assessment for BDD-TEL. The initial assessment interview was conducted face-to-face by a psychotherapist with appropriate pandemic safety measures. Questionnaires were completed and returned immediately after the interview. In addition, in this preliminary face-to-face session, patients received their workbook. After this initial assessment, BDD-TEL commenced; it included 12 weeks of 30 min telephone treatment sessions, followed by a face-to-face post-treatment assessment. The patients’ risk of suicide was assessed in each session.

## 3. Results—Case Series

The following section describes four case reports treated with BDD-TEL. After the presentation of the individual case reports, Table 2 provides an overview of the reported symptoms at baseline and treatment outcomes.

### 3.1. Case 1

Background: Case 1 was a mechanical engineer approximately fifty years old who had been in various treatment programs over the last 30 years for different mental health problems, especially social phobia and depression. He expressed that he had never been diagnosed or treated for BDD, although in his opinion, BDD was the cause of his other mental health problems. SCID revealed comorbid BDD, major depression, social phobia, and binge eating. He was divorced and had two children, but recently had started living alone. He worked full time but was on leave due to the measures in place during the COVID-19 pandemic, which might have led to an increase in body dysmorphic symptoms. He had experienced appearance-related concerns since he was a teenager; in his impression, his BDD had not yet been properly treated. At the beginning of treatment, the appearance-related concerns related to the appearance of his chin, which he felt was underdeveloped. He felt his appearance was “like a monster” and could barely stand his reflection in the mirror. The appearance-related worries limited his activities, especially in social situations; face-to-face contact was difficult for him, and he avoided social situations.

Treatment outcome: Through BDD-TEL, he was able to better understand his own BDD and categorize his symptoms as such. In Modules 1 and 2, he realized that his problem was his safety and avoidance behaviors rather than his appearance. In Module 3, he recognized substantial black-and-white thinking in himself. Through planned mirror exposures (Modules 4 and 5), he was able to find a way to deal with his own reflection (especially looking at his whole face). Therefore, he was able to participate in social life again and attended a photography course as well as a self-help support group. There was clinically relevant symptom improvement in BDD but not in depression or anxiety. After treatment with BDD-TEL, he still felt insecure in social situations, but he knew which methods and skills (especially exposure) could help him and that avoidance behavior would maintain or worsen his insecurity. After BDD-TEL, he was informed that some of his mental health conditions persisted and was therefore offered further CBT, but he wanted to continue practicing the methods and skills on his own. He was advised to monitor his symptoms carefully and reach out if the symptoms worsened.

### 3.2. Case 2

Background: Case 2 was a student at the end of his studies, in his mid-twenties. He was single and lived with his parents. He reached out to the outpatient clinic due to BDD symptoms; it was his first contact with psychotherapeutic treatment. SCID revealed BDD comorbid with major depression. His main concerns were his ankle, which—to him—seemed to be disfigured after an accident, and a scar on his face. As a safety behavior, he used many concealers to cover the small scar on his face. The facemasks during the COVID-19 pandemic made it easier for him to participate in social situations. However, they reinforced thoughts of his appearance without a mask. In addition, he showed avoidance behavior regarding his ankle by not wearing shorts or sandals. His depressive symptoms worsened as social withdrawal and a decline in positive activities increased.

Treatment outcome: In the first sessions, behavioral activation was conducted in parallel with psychoeducation (Modules 1 and 2). In particular, planning physical exercise breaks in his daily routine of writing his master’s thesis improved his depressive symptoms (mood and daily drive). In Module 3, he realized that he was using selective attention combined with a tendency to perfectionism. Whereas the benefits of selective attention were helpful to discuss, perfectionistic attitudes toward his appearance, his work and his life in general were more difficult to address. However, he realized that these attitudes could be a stumbling block for him, especially in evaluation situations. He learned how thoughts, feelings, and behaviors are inter-related and especially the effect of safety and avoidance behaviors in maintaining his symptoms. This motivated him, after exposures (e.g., riding a train in sandals) were planned and discussed together (Modules 4 and 5), to independently plan and carry out further exposures. The patient was very grateful for the workbook, as he was able to continue working independently between sessions. The cognitive restructuring methods and exposures related to the ankle were central to his progress. Improvements in BDD symptoms and depressive symptoms were reported; after BDD-TEL, he did not meet any criteria for mental disorders.

### 3.3. Case 3

Background: Case 3 was a female student in her late twenties who was living with her partner. She contacted the outpatient clinic because of its specialization in BDD. She had no prior psychotherapeutic experience in her adult life and reached out to the clinic for treating her BDD symptoms. She saw various flaws in herself, especially in her face; this prompted her to often ritualistically stand in front of the mirror and pull on her face in a certain way with her fingers and to make specific expressions while looking at the reflection. In addition, she reported avoidance behaviors: because of the shape of her chest and the color of her skin, she no longer went to the pool. She used to have many interests (e.g., drawing) that she no longer pursued. SCID I revealed major depression comorbid with BDD.

Treatment outcome: The patient was very grateful for the provided structure and was very motivated to perform all the exercises. Within the first two modules, she got to know herself well through the BDD diary, and her self-insight increased. She found that the BDD diary was too negative; therefore, she started a gratitude diary. She also used self-affirmations “I am beautiful” or “I am ok” and told herself what incredible things her body could do. Modules 4 and 5 helped her to understand the importance of exposures, and she consecutively undertook and performed more difficult ones. Moreover, she went to a public swimming pool without any safety behavior. Overall, there was an improvement in her insight as well as in symptoms of depression and BDD; after BDD-TEL, she did not meet any criteria for mental disorders.

### 3.4. Case 4

Background: Case 4 was a single receptionist in her thirties who was on leave from work and living alone. SCID revealed major depression and hypochondria comorbid with BDD. She had already undergone various treatments for different disorders and had ambivalent illness insight. She contacted the BDD specialty outpatient clinic to be treated specifically for BDD. The focus of her concern was her nose, which had persisted since she was a teenager and caused her to have multiple plastic surgeries.

Treatment outcome: The patient did not complete BDD-TEL. After two sessions of BDD-TEL, it became apparent that the patient’s other health-related fears were clearly the focus of concern. These led to severe panic attacks and simultaneous high arousal with racing thoughts about suicide (questioning her life in case of a diagnosis). The patient was recommended for inpatient treatment, as weekly sessions were not enough to break this vicious cycle. It is possible that a comorbid disorder (e.g., generalized anxiety disorder) was not recognized during the diagnostic session to the extent that the treatment for BDD interfered; additionally, her insight at preassessment was the poorest, and her symptoms were the most severe.

## 4. Discussion

Telephone-administered CBT has been shown to be successful in the treatment of other disorders and problem situations (e.g., [20,29]); however, to date, there is no research on BDD. During the COVID-19 pandemic and the required containment measures, BDD-TEL was developed in reference to the core elements of CBT. The findings suggest that the intervention was effective in reducing BDD symptoms. Three patients reported BDD symptom reduction, as well as an improvement in insight and a decrease in depressive symptoms in some. Two patients did not meet any criteria of any disorder after treatment. In one patient, the therapy was not successful, and another treatment was recommended. Compared to BDD-NET [12,13,14,15], BDD-TEL offers a treatment with more minutes of human contact. In the case series presented, BDD-TEL has shown promising results that align with low-intensity treatment options for OCD [19]. Since this is only a case series, no generalizations to the population as a whole or comparisons to BDD-NET can be made. The next step would be to test the efficacy of BDD-TEL in a larger sample, considering different comorbidities and severities, to be able to determine the adequate indication/target population and to optimize the advantages of remote treatment (e.g., no travel necessary) or possible difficulties (e.g., suicidality assessment). Additionally, specific predictors of treatment response might be identified. Further, it would be interesting to compare the efficacy (for BDD symptoms, depressive symptoms) and insight, as well as adherence, acceptance and drop-out rates between different treatment modalities. As BDD-TEL is of interest in stepped-care programs, predictors of more intensive treatment would be of interest in future studies. Furthermore, the intervention was well accepted in the patients, especially during the COVID-19 pandemic, as they reported during the sessions, which is in line with research on BDD and COVID-19 [22,23]. Post-pandemic BDD-TEL could be of interest to reduce some treatment barriers (e.g., distances to travel), while still building a more human working alliance than in internet interventions. Even if patients cannot leave the house (e.g., due to diseases on the one hand, but also due to avoidance behaviors), BDD-TEL could be an important first step. However, if it is due to avoidance behavior, face-to-face therapy would be indicated as soon as possible.

Some patients also mentioned topics that they wanted to be included in the workbook and modules that they found difficult: As in BDD-NET [12,13,14], the modules focusing on exposure were considered the most difficult. These impressions (e.g., the BDD diary seemed to be negative for Case 3) could lead to the improvement of the workbook before it is scientifically evaluated. The acceptance and feasibility of BDD-TEL are questions that can be addressed in future scientific studies. As a comorbid depression with BDD is common, adding behavior activation could be beneficial at the beginning of BDD-TEL. As in Enander et al. [12,13,14], the largest drop-out was during the modules focusing on exposure, thus, starting with behavioral activation could help motivate patients to continue their progress through further treatment. Video-conferencing tools could be beneficial, especially during Modules 4–5. However, as some patients mentioned having issues with seeing their own reflection in the screen and themselves in the video-conferencing tool, it would need to be addressed with caution to avoid adding another treatment barrier.

## 5. Conclusions

BDD-TEL is a possible addition to the low-threshold treatment of BDD [12,13,14,15,16,17], as it is a remote treatment, without the complications of reflections on cell phones or screens and with slightly more contact with a therapist. Like all low-threshold therapy services, it could potentially be useful in stepped-care but would need scientific investigation.

## Figures and Tables

**Table 1:** Structure and content of BDD-TEL “Being able to live with the mirror”.

Module	Content	Work Sheets
Module 1 *«Questions and answers about body dysmorphic disorder»*	Psychoeducation on CBT (thoughts feelings, behaviors), BDD (e.g., specific symptoms, prevalence, risk factors, body image), on the telephone setting and on CBT for BDD.	Preparation and follow-up for weekly telephone session, body image and optical illusions, my avoidance and safety behaviors, my diary (self-observation task)
Module 2 *«A psychological explanatory model of BDD»*	Explanatory model: triggering and sustaining conditions, specific thoughts and interpretation traps, unpleasant feelings (e.g., shame) and their implications for our behavior (safety and avoidance behavior).	Preparation and follow-up for weekly telephone session, theory A vs. B, my explanatory model of BDD, my diary
Module 3 *«Thoughts and interpretation traps»*	Psychoeducation on interpretations vs. facts, lemon exercise, interpretation traps (e.g., all-or-nothing, selective attention), helpful vs. unhelpful thoughts, if-then chains.	Preparation and follow-up for weekly telephone session, my interpretations, my if-then chain, my diary
Module 4 *«Introduction to exposure and reaction prevention»*	Short and long-term consequences of avoidance and safety behaviors, psychoeducation on exposure and reaction prevention (habituation, exposure hierarchy, short and long-term consequences, side effects, different possibilities), setting a goal.	Preparation and follow-up for weekly telephone session, my goals (emotional, behavioral, long-term), my exposure hierarchy, planning my exposure and reaction prevention
Module 5 *«More on exposure and reaction prevention»*	Planning and performing exposure and reaction prevention, notes on implementation of exposure and reaction prevention (repetitions, time required, integration into everyday life, difficulties), rumination.	Preparation and follow-up for weekly telephone session, my exposure and reaction prevention
Module 6 *«Value-oriented directions and goals»*	Quality of life and being content, values, value-oriented directions and goals, balance of positive and negative activities, different areas of life.	Preparation and follow-up for weekly telephone session, what is important in my life?, my funeral, my value-oriented directions and goals, my weekly schedule, continuing with exposure and reaction prevention
Module 7 *«Difficulties and setbacks»*	Frequent difficulties with exposures (e.g., lack of motivation, feeling better or worse), dealing with setbacks.	preparation and follow-up for weekly telephone session, continuing with exposure and reaction prevention
Module 8 *«Summary and outlook»*	Summary and outlook, what is next?, how to maintain the success of the treatment, setbacks and relapses, high risk situations, early warning signs.	Preparation and follow-up for weekly telephone session, my relapse prevention plan, my already achieved goals, my already achieved goals, my high-risk situations and early warning signs

**Table 2:** Reported symptoms and treatment outcomes.

Case	Pre-Treatment	Post-Treatment	Changes
**BDD Symptoms (FKS) ^1^**			
1	33	21	36.4%
2	41	15	63.4%
3	45	15	66.7%
4	48		
**Depressive Symptoms (PHQ-9) ^2^**			
1	13	13	0
2	14	2	85.7%
3	17	1	94.1%
4	22		
**Anxiety Symptoms (GAD-7) ^3^**			
1	5	4	20%
2	16	2	87.5%
3	14	3	78.6%
4	16		
**Functional impairment (additional item PHQ-9) ^1^**			
1	2	1	50%
2	3	0	100%
3	3	1	66.7%
4	3		
**Insight (BABS) ^4^**			
1	10	3	70%
2	8	0	100%
3	12	3	75%
4	17		
**Met DSM ^5^ criteria for disorders? (SCID) ^6^**			
1	Yes	Partial	-
2	Yes	No	-
3	Yes	No	-
4	Yes	-	-

Note: FKS ^1^ = “Fragebogen Körperdysmorpher Symptome”, questionnaire for BDD symptoms (Cutoff: 14), PHQ-9 ^2^ = Patient Health Questionaire-9 (mild depression: 5–9, moderate depression: 10–14, severe depression 15–27), GAD-7 ^3^ = General Anxiety Symptoms-7 (mild anxiety: 5–9: moderate anxiety: 10–14, severe anxiety: 15–21), BABS ^4^ = Brown Assessment of Beliefs Scale (0–3: excellent insight, 4–7 good insight, 8–12: sufficient insight, 13–17: poor insight, ≥18: no insight). DSM ^5^ = Diagnostic and Statistical Manual of Mental Disorders, SCID ^6^ = Structured Clinical Interview for DSM.
